# Physiological and Perceived Stress, Anxiety, Depression and Burnout Among ICU Staff During the COVID‐19 Pandemic in Sweden—An Observational Study

**DOI:** 10.1111/aas.70081

**Published:** 2025-06-23

**Authors:** Henrik Andersson, Tomas Faresjö, Helén Didriksson, Carina Jonsson, Gunilla Gagnö, Michelle S. Chew

**Affiliations:** ^1^ Department of Anaesthesia and Intensive Care, Biomedical and Clinical Sciences Linköping University Linköping Sweden; ^2^ Department of Health, Medicine and Caring Sciences, General Practice Linköping University Linköping Sweden; ^3^ Department of Perioperative Medicine and Intensive Care Karolinska University Hospital Huddinge Sweden

**Keywords:** anxiety, burnout, COVID‐19, depression, stress

## Abstract

**Purpose:**

To measure chronic, physiological stress using hair cortisol concentrations (HCC) among ICU staff working during the COVID‐19 pandemic in Sweden, and to evaluate its relationship with perceived stress (PS), anxiety, and depression, and burnout.

**Materials and Methods:**

HCC was measured in hair samples of 274 individuals. We tested for differences between females versus males, nurses versus non‐nursing, and ordinary versus deployed staff. PS, anxiety, depression, and burnout were measured using validated questionnaires.

**Results:**

Median HCC was 32.6 pg/mg [IQR 21.3–62.3] reflecting physiological stress over 3 months. There were no differences due to sex, profession, and deployment status. Anxiety was detected in 19% and depression in 4% of participants. PS score (23.5 [18.25–29.0]) was higher than previously reported normative data. Burnout was driven by low personal accomplishment. No correlations were found between HCC and PS, anxiety, depression, and burnout.

**Conclusions:**

Chronic, physiological stress measured as HCC was not different due to sex, profession or deployment status. Anxiety and burnout were frequent and depression was reported by a minority. HCC did not correlate with PS, anxiety, depression or burnout.

## 1 Introduction

It is now 5 years since the onset of the pandemic and multiple studies have documented increased rates of chronic stress, insomnia, fatigue, anxiety, and depression and burnout [1, 2, 3, 4, 5, 6, 7, 8]. Female workers and nurses appear to be disproportionately affected, with increased levels of burnout reported during the pandemic [5, 7]. Chronic stress and burnout are major drivers behind the unprecedented global shortage in health care workers today [9].

Validated instruments to assess mental health such as the hospital anxiety and depression scale (HADS) [10] and the perceived stress scale (PSS) [11] together with established questionnaires such as the Maslach Burnout Inventory—Human Services Survey (MBI‐HSS) [12] can provide insights into the experiences of ICU staff and how anxiety, depression, occupational exhaustion, and burnout are linked [13, 14, 15].

However, none of these instruments reflect the biological effects of stress, which may be of consequence when trying to establish a pathophysiological link between self‐reported or experienced stress and biological function. Physiological effects of chronic stress include downregulation of the hypothalamic–pituitary–adrenocortical axis (HPA axis) [16] with increased levels of catecholamines and glucocorticosteroids such as cortisol in blood and tissues, potentially giving rise to hypertension, coronary artery disease, obesity, and poor mental health [17, 18, 19]. The long‐term consequences of chronic stress are significant and have been documented in populations exposed to economic crises [20, 21]. Recently, an association between burnout and elevated cardiovascular risk was demonstrated among healthcare professionals working during the pandemic [22]. Therefore, evaluation of the physiological effects of stress is relevant.

Plasma cortisol is a biomarker for stress and can be measured in saliva, blood, and urine. However, these measurements only reflect stress at the time of sample collection. Cortisol levels can also be determined in hair and reflect the allostatic load of chronic stress over longer periods of time [23, 24, 25, 26, 27]. Hair grows approximately 1 cm per month, and the measurement of cortisol levels in hair reflects stress levels during the time of hair growth. This makes it possible to study physiological stress retrospectively. Physiological stress may be reliably measured up to 6 months prior to sample collection [24].

Little is known about how the COVID‐pandemic has contributed to chronic, physiological stress within anaesthesiology and intensive care, which has contributed to a disproportionate proportion of health care workers at the frontline.

The aim of this study was to measure chronic, physiological stress using hair cortisol concentrations (HCCs) as a biomarker among ICU staff involved in the care of critically ill patients during the COVID‐19 pandemic in Sweden; and evaluate its relationship with perceived stress, anxiety and depression, and burnout. We compared males versus females, nurses veresus non‐nursing, and ordinary versus deployed staff.

## 2 Methods

We conducted a two‐centre cross‐sectional study, with data collection during the period October–December 2021. The main outcome variables were chronic, physiological stress during the last 3 months, measured as HCC, and self‐reported perceived stress, anxiety, depression, and burnout. Ethical approval was obtained by the Swedish Ethical Review Authority (2021‐04272).

### 2.1 Participants

All personnel caring for COVID patients in the two ICUs in Region Östergötland County, Sweden during the COVID‐19 pandemic between March 2020 and December 2021 were eligible for inclusion. This included physicians, nurses, nurse assistants, physiotherapists, and administrative staff. They were contacted by mass email and information during staff meetings and invited to participate in the study. Written informed consent was provided by all participants.

Exclusion criteria were disorders of the HPA‐axis (e.g., Addison's disease, Cushing's disease) [27], chronic high‐dose corticosteroid therapy, and an inadequate length of hair at the back of the scalp (< 3 cm).

### 2.2 Demographic Information

For all participants, we recorded sex, age, profession, ordinary place of work, and place of work during the COVID‐19 pandemic. The latter was required to distinguish between ordinary and forcibly deployed ICU staff during the pandemic.

### 2.3 Analysis of Hair Cortisol Levels

A hair sample was cut with scissors as close to the scalp as possible from the posterior vertex, as this is the location with the lowest intra‐individual variation in cortisol concentration [28]. The 3 cm of hair closest to the hair roots was analysed, reflecting stress exposure during the last 3 months. Cortisol was measured in methanol extracts of hair using an inhouse competitive radioimmunoassay as described previously [29]. Briefly, hair was weighed and at least 5 mg was used for the analysis. The samples were minced and cortisol was extracted in methanol, lyophilized, and then refrigerated for storage. Cortisol was measured using a radioimmunoassay. At 10 nmol/L, the intra‐ and interassay coefficients of variation were 6.1% and 9.3%, respectively, and the lower limit of detection was 1 nmol/L.

### 2.4 Questionnaires

At the time of hair sampling, all participants answered the PSS, HADS, MBI‐HSS and AWS. The PSS is a self‐reporting tool designed to evaluate how stressful the respondent has experienced situations in their life during the last month and determine to what degree the participants found their life unpredictable, overloading, and uncontrollable [11]. The translated and validated Swedish version of the PSS was used [11, 30]. The PSS is not a diagnostic instrument and there are no score cut‐offs.

HADS is a validated 14‐item questionnaire [10] to detect states of anxiety and depression in non‐psychiatric hospital clinics. The questionnaire consists of one part each for the measurement of anxiety and depression, and participants are scored as normal (< 7 points), borderline case (7–10 points), and case (> 10 points).

MBI‐HSS (MP) is a validated 22‐item questionnaire assessing experienced professional burnout among healthcare professionals. The questionnaire assesses three characteristics of burnout: emotional exhaustion (EE), depersonalization (DP), and personal accomplishment (PA) [12]. A combined burnout score was calculated by the formula EE + DP‐A. For EE, a score < 19 was defined as low, 19–26 as moderate, and > 26 as high. For DP, a score < 6 was defined as low, 6–9 as moderate, and > 9 as high. For PA, a score < 29 was defined as low, 29–39 as moderate, and > 39 as high. For the combined burnout, a score < −21 was defined as low, −21 to −9 as moderate, and > −9 as high level of burnout.

AWS is a 28‐item questionnaire assessing sources of burnout [31]. The scale ranges from 1 (strongly disagree) to 5 (strongly agree) and is divided into six key domains:

Workload: When job demands exceed human limits.

Control: Perceived capacity to influence decisions that affect work.

Reward: The extent to which rewards—monetary, social, and intrinsic—are consistent with expectations.

Community: The overall quality of social interaction at work, including issues of conflict, mutual support, closeness, and the capacity to work as a team.

Fairness: The extent to which decisions at work are perceived as being fair and people are treated with respect.

Values: The motivating connection between the worker and the workplace that goes beyond the utilitarian exchange of time for money or advancement.

All scores were summed in accordance with the instructions for each questionnaire [10, 11, 12, 31], with HADS, MBI, and AWS being divided into their respective subdomains.

### 2.5 Statistical Analysis

Differences between groups were analyzed using Chi square, Mann Whitney‐U test, or Kruskal Wallis tests as appropriate. The following subgroups were prespecified for comparison: males versus females, nurses versus non‐nursing staff, and ordinary versus deployed staff. In an exploratory analysis, we also compared staff that held leadership positions versus those who did not. To examine whether a higher HCC correlated with higher scores on the various questionnaires, Spearman correlation analysis was used. Participants who had missing data points from the questionnaires (e.g., due to skipping an item) were excluded from the analysis of that specific questionnaire. There were no missing sociodemographic data. Statistical analyses were performed using the Statistical Package for the Social Sciences (SPSS, ver. 28) software. A *p* value of ≤ 0.05 was considered statistically significant.

## 3 Results

A total of 453 ICU staff were eligible for the study and 274 (61%) participated. The median age was 46 years and 85% were female (Table 1). The largest professional group was nurses (44%) followed by nurse assistants (41%), physicians (8.8%) and other staff (administrators, physiotherapists, laboratory technicians) (5.8%). 4.7% had a leadership position, and 36% were not ordinary ICU staff but deployed to ICUs during the pandemic.

**TABLE 1:** Characteristics of the study population.

Age (years, IQR)	46 (36‐54)
Sex female (*n*, %)	233 (85%)
Profession	
Physician	24 (8.8%)
Nurse	121 (44%)
Nurse assistant	113 (41%)
Other	16 (5.8%)
Leadership position	13 (4.7%)
Non‐ordinary staff deployed to ICU	99 (36%)

*Note: n* = 274.

### 3.1 Hair Cortisol Concentrations

The median HCC was 32.6 pg/mg (IQR 21.3–62.3). The median HCC for females was 31.6 pg/mg (21.1–61.7) versus 37.9 pg/mg (22.1–92.7) for males (*p* = 0.315). No significant differences were found when comparing nurses versus non‐nursing staff, ordinary versus deployed staff, and staff with and without leadership positions (Figure S1).

### 3.2 Hospital Anxiety and Depression Scale

**FIGURE 1:**
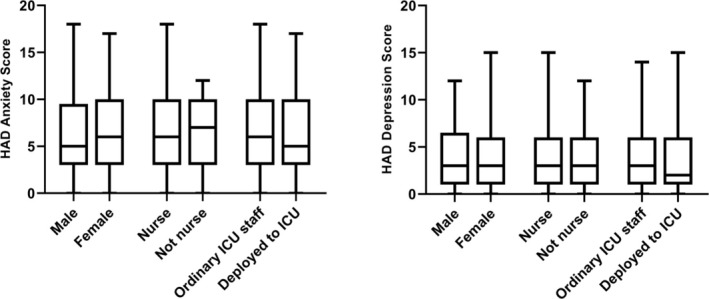
Anxiety and depression stratified to sex, profession (nurse vs. not nurse), and ordinary place of work (ordinary vs. deployed staff). *n* = 273.

The median scores for the HADS‐anxiety subscale and HADS‐depression subscale were 6 (IQR 3–10) and 3 (IQR 1–6) respectively. When defined as a subscore of > 10%, 19% of the participants suffered from anxiety and 4% from depression (Table 2). No significant differences were found when comparing male versus female, nurses versus non‐nursing staff, and ordinary versus deployed staff (Figure 1). There were no differences between staff with and without leadership positions (Figure S2).

**TABLE 2:** Hospital anxiety and depression scale.

	Anxiety	Depression
No anxiety/depression (score < 7)	156 (57%)	219 (80%)
Mild/moderate (score 7–10)	64 (23%)	43 (16%)
Anxiety/depression (score > 10)	53 (19%)	11 (4%)

*Note: n* = 273.

### 3.3 Perceived Stress Scale

**FIGURE 2:**
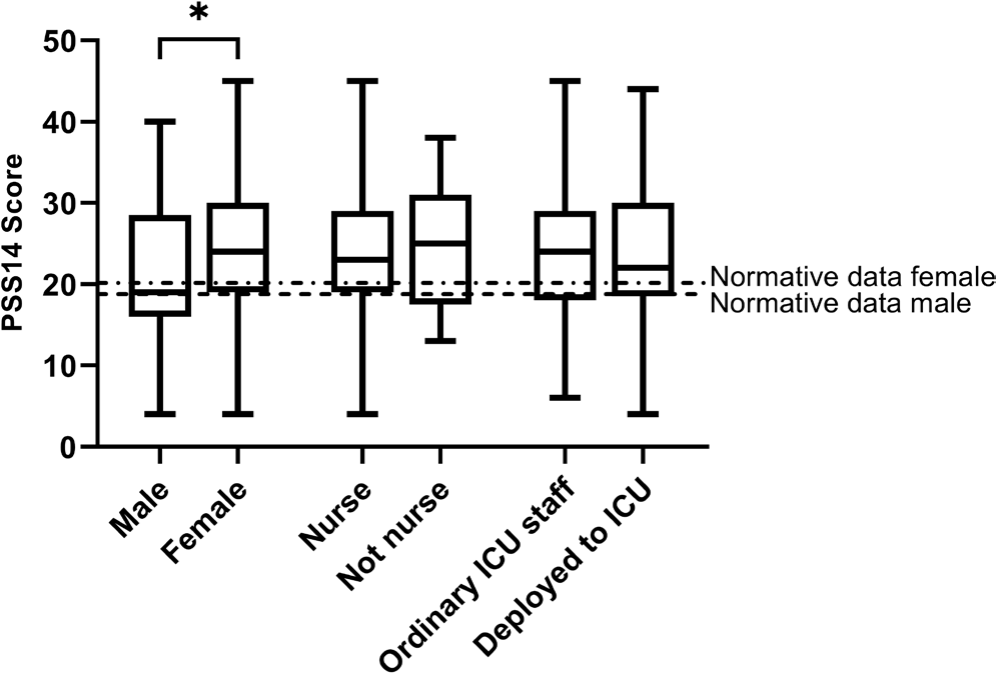
Perceived stress stratified to sex, profession (nurse vs. not nurse), and ordinary place of work (ordinary vs. deployed staff). Comparative levels for PSS for the general male (18.8 [SD 6.9]) and female (20.2 [SD 7.8]) population in the USA are indicated (Cohen 1988). *n* = 274.

The median score was 23.5 (IQR 18.25–29.0). Females had significantly higher perceived stress than males (24.0 [19.0–30.0] vs. 19.0 [16.0–28.5], *p* = 0.021) (Figure 2). Comparative levels for PSS for the general male and female population in the USA [32] are also indicated. No significant differences were found between nurses and non‐nursing staff, ordinary versus deployed staff (Figure 2) or between staff with and without leadership positions (Figure S3).

### 3.4 Maslach Burnout Inventory

High levels of overall burnout were identified in 38.3% of the cohort (Table 3). A high degree of EE was demonstrated in 25%, 6% reported a high degree of DP and 20% had a low sense of PA.

**TABLE 3:** Maslach Burnout Inventory.

MBI‐HSS (MP)	EE	DP	PA	Combined
Low	112 (42%)	193 (74%)	51 (20%)	84 (33.2%)
Moderate	87 (33%)	52 (20%)	78 (30%)	72 (28.5%)
High	66 (25%)	17 (6%)	129 (50%)	97 (38.3%)

*Note:* Data presented as number and % of participants with low, moderate and high levels of burnout in each subcategory of the Maslach burnout inventory. *n* = 265 (EE), 262 (DP), 258 (PA) and 253 (combined). For EE, a score < 19 was defined as low, 19–26 as moderate and > 26 as high. For DP, a score < 6 was defined as low, 6–9 as moderate and > 9 as high. For PA, a score < 29 was defined as low, 29–39 as moderate and > 39 as high. For the combined burnout, a score < −21 was defined as low, −21 to −9 as moderate and > −9 as high.

**FIGURE 3:**
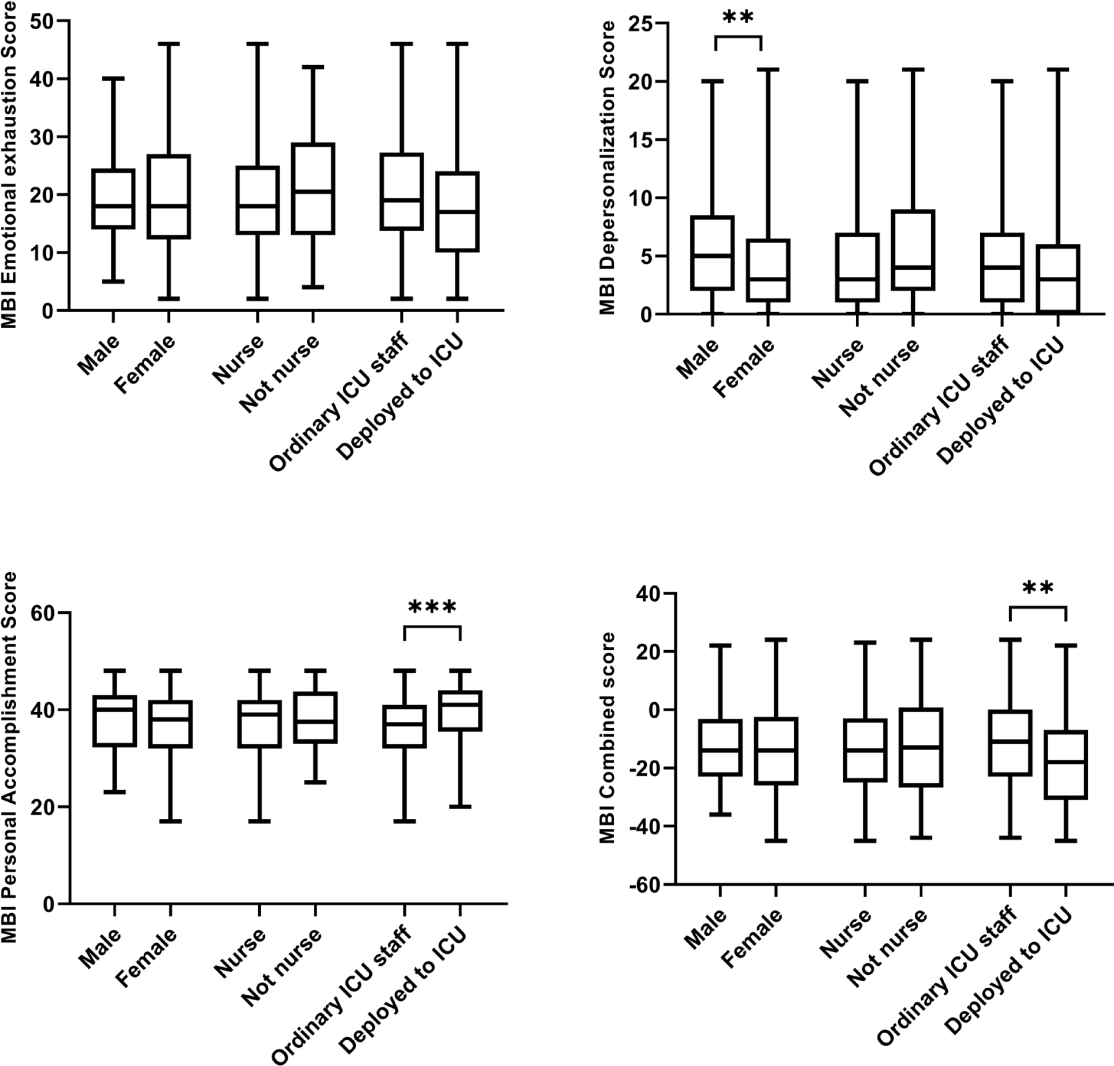
Emotional exhaustion, depersonalization and personal accomplishment stratified to sex, profession (nurse vs. not nurse), and ordinary place of work (ordinary vs. deployed staff). *n* = 265 (EE), 262 (DP), 258 (PA).

Males had a higher degree of DP than females (Figure 3) but there were no differences due to sex for EE and PA. There were no significant differences between nurses and non‐nursing staff in any of the domains. Staff that were deployed from other areas to work in ICU during the pandemic had lower levels of overall burnout (−18 [−31–(−7)] vs. −11 [−23–0], *p* = 0.003), with higher levels of PA. Staff with leadership positions had higher scores for EE (31.0 vs. 18.0, *p* = 0.0028), and DP (4.0 vs. 3.0, *p* = 0.0373), but similar levels of PA compared to staff without leadership positions.

### 3.5 Area of Worklife Survey

**FIGURE 4:**
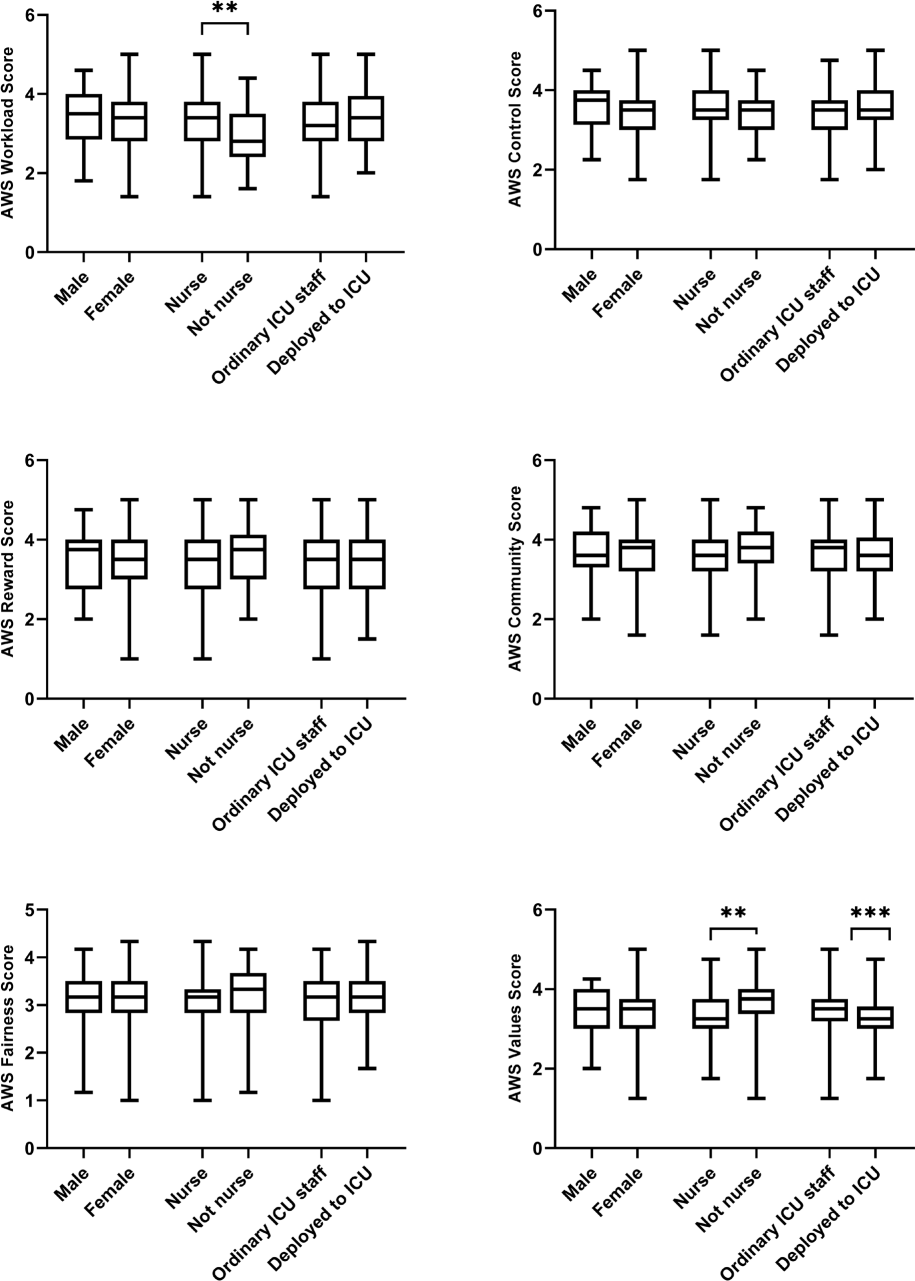
Area of worklife survey. The key domains stratified to sex, profession (nurse vs. not nurse), and ordinary place of work (ordinary vs. deployed staff).

There were no differences between female and male staff in any of the AWS domains (Figure 4). Nurses scored higher than non‐nursing staff in the workload domain (3.4 [2.8–3.8] vs. 2.8 [2.4–3.5], *p* = 0.001) while non‐nursing staff scored higher than nurses in the values domain (3.75 [3.375–4.0] vs. 3.25 [3.0–3.75], *p* = 0.002). Higher scores in the values domain were also seen in ordinary versus deployed staff (3.5 [3.188–3.75] vs. 3.25 [3.0–3.563], *p* = 0.0001).

### 3.6 Association Between HCC and Mental Health

Spearman's correlation analysis showed no correlation between hair cortisol levels and the scores from any of the instruments HADS, PSS, or MBI.

## 4 Discussion

HCCs reflecting chronic, physiological stress were measurable in this population of ICU staff working during the COVID‐19 pandemic. However, there was no relationship between HCC and self‐reported anxiety, depression, perceived stress, and burnout. High scores for anxiety were demonstrated in every fifth ICU staff member during the COVID pandemic, and perceived stress was higher than in previously described normative data from a general American population [32]. Burnout was common and occurred in 38.3% of Swedish ICU staff.

In investigating the relationship between perceived and physiological stress, our interpretation was complicated by the fact that PSS has no cut‐offs for low, medium, and high levels of perceived stress. The median PSS score for all participants was 23.5, comparable to a study among Swedish university students with a mean score of 24.4 (SD 8.0) [33] but higher than the mean score in the general population in the USA (19.62 (SD 7.49)) [32]. Nevertheless, we found no significant correlation between PSS and HCC in our study, in line with a recent study among anaesthesiologists that also found no correlation between hair cortisol levels and self‐reported psychological stress [34]. These findings highlight difficulties in the interpretation of stress levels. Perceived stress is inherently a subjective experience and may or may not be related to biochemical concentrations. Although the PSS requires participants to reflect on their life during the last month, there is likely to be recall bias for events closer to the time of the survey. Serum cortisol measurements are limited by diurnal variation and represent only shorter‐term events. Whereas HCC is indicative of chronic changes, it may be too insensitive to reflect acute changes. Further research will improve our understanding of how to best use HCC.

There was no significant correlation between HCC and scores on the HADS questionnaire. The relationship between HCC and anxiety and depression remains unclear with one study demonstrating increased levels in major depression and decreased levels in patients with anxiety [35]. However, another study [36] showed that patients with major depression had lower HCC compared to healthy controls, and patients with general anxiety disorder had no differences in HCC compared to the control group. We did not find a relationship between HCC and the anxiety‐specific or depression‐specific scores of the HAD.

Our findings regarding anxiety, depression, and burnout are consistent with previously reported data in ICU staff [5, 6, 7, 37]. Nevertheless, the levels reported in this study seem to be lower than those previously reported. High scores for anxiety were demonstrated in every fifth ICU worker in our population. Depression was uncommon (4%), with approximately the same prevalence as in the normal population [38], suggesting that our study cohort seemingly did not suffer from more depression due to the pandemic. This was a surprising but somewhat reassuring finding, given the significant burden of emotional and physical challenges imposed on ICU staff during the COVID‐19 pandemic. Although the prevalences of anxiety and depression in our population appear low compared to a recent, large European survey [5], this discrepancy may be explained by the use of different cutoff levels. When applying the same cutoffs (i.e., HAD scores > 7), the prevalences of anxiety and depression in our population were similar to the European study at 42% and 20%, respectively.

Burnout was common and occurred in 38.3% of the population, with 25% reporting EE, and 20% reporting low PA. In contrast, depersonalisation occurred only in a small proportion of staff. This prevalence was unexpectedly low compared to the 52% reported by Azoulay et al. [5] where all three domains of EE, DP and PA were generally equally affected. The lower proportion of staff reporting depersonalisation and low PA are also at odds with those reported in a recent meta‐analysis [39] where burnout in these domains was found in 30%–40% of ICU physicians and nurses. We cannot explain these findings, and do not have any previous Swedish data in ICU staff to compare with. We can only speculate about whether Swedish workplace practices may have contributed to these differences.

Poor mental health is not a new phenomenon among ICU staff [37, 39], and the negative impact of the COVID‐19 pandemic including increased levels of burnout has been previously documented [4, 6, 7, 39], with nurses being disproportionally affected [7, 39]. Although we did not measure prepandemic levels of burnout in our study, we can confirm that a large proportion of Swedish ICU staff are exposed to burnout, without any apparent differences between males and females, and between nurses and non‐nursing staff. There were too few samples within each professional group to allow a robust comparison between nurses' aides, registered nurses, physicians and other healthcare professionals. The intersectionality between profession and gender is relevant, since both nurses and female sex are known risk factors for burnout [4, 5, 7, 40, 41], however since we had a very limited sample size of male participants (*n* = 41) and other professions (24 physicians, 16 other), we were unable to study this question. Thus, we suggest that future research should investigate interprofessional differences and the intersectionality between gender and profession.

Among ICU staff with high levels of burnout, the main driver was a low PA. This differs somewhat from previous studies where higher proportions of EE and DP were noted [5, 39]. We were unable to investigate the possible reasons behind this and hypothesize that organizational factors as well as specific characteristics of the Scandinavian population may have contributed. Two other interesting observations emerged in our data. Firstly, burnout was particularly high among staff in a leadership positions, with differences in the subdomains of EE and DP. No differences were noted between any of the other subgroups by profession, sex, or ordinary place of work. Although these observations were only based on a limited number of participants (*n* = 13 in leadership positions) and cannot be considered robust, they are in line with a recent study of Swedish healthcare managers during the first wave of the COVID‐19 pandemic [42] and raise questions of whether focused efforts should be directed at this particular group of healthcare workers that experience different pressures for performance and accountability. The second interesting observation was that burnout was not more common among deployed staff that made up 36% of the participants in this study. Contrary to our expectations, deployed staff reported a higher level of PA. This observation contradicts a previous qualitative study [43] where deployed nurses generally reported negative experiences. Deployment to unfamiliar work situations is important to address not least for potential strategies to build resilience and has hitherto been poorly studied. We can offer some possible explanations for our findings. Firstly, the vast majority of this group comprised specialist nurse‐anaesthetists with pre‐existing airway and haemodynamic monitoring skills and had been exposed to the care of the critically ill under anaesthesia prior to the pandemic. There was also some understanding of the “culture” of intensive care since anaesthesia and intensive care are a combined specialty in Scandinavia. Secondly, all staff deployed to work in the two ICUs from other areas were given a 2–3‐day training program in anticipation of a “wave” of COVID‐19 patients. This may have mitigated some of the difficulties experienced by nurses who did not have prior ICU experience.

This study has several notable limitations. The study was conducted at two hospitals in Sweden, and the findings reflect only experiences in these institutions and may not be generalizable. The present study was cross‐sectional in design, so it is not possible to evaluate the longitudinal effects of physiological and perceived stress, anxiety, and depression, and burnout. It is also conceivable that the results may have been different with longer exposure to the COVID‐19 pandemic. Secondly, we were only able to collect data from existing staff. ICU staff who may have worked during the pandemic but resigned prior to the start of the study could not be sampled, and this may have induced an attrition bias. There is also no previous data on burnout, anxiety, and depression among Swedish ICU healthcare workers with which we can contextualize our findings. Similarly, there is no previous data available on hair cortisol measurements in ICU staff.

Our study relied on voluntary participation. We enrolled approximately 61% of all ICU staff working during the pandemic but were unable to collect even baseline data that may have alluded to differences between the sampled and non‐sampled populations. Perceived stress, anxiety, depression, and burnout were measured using self‐reported questionnaires with limitations due to recall‐ and subject bias. Additionally, PSS measures the perceived stress experienced over the previous month, whereas HADS and MBI evaluate the anxiety, depression, and burnout currently experienced, hence not accounting for previously experienced anxiety, depression, or burnout symptoms.

The data for this study was collected during October–December 2021, which is approximately 2 years after the start of the Swedish COVID‐19 pandemic. We did not assess the contribution of continued exposure to working in ICUs. This may have had a bearing on the physical and mental wellbeing of the ICU staff and may in part explain current unprecedented shortages in ICU workforce.

Nevertheless, our findings offer a few added insights into ICU staff wellbeing during the pandemic. Strengths of this study include its multiprofessional sampling and the multidimensionality of measurements including both biochemical and psychological components. Our results do not support that HCC may be used as a simpler method for screening or monitoring suboptimal mental well‐being; however, much more work is needed to better understand the relationship between chronic, physiological stress and psychological outcomes, including comparisons with other indicators such as staff turnaround.

## 5 Conclusions

Chronic, physiological stress was measurable as HCCs among ICU staff without differences due to sex, profession and ordinary versus deployed status. One in five ICU staff working during the COVID‐19 pandemic experienced high levels of anxiety, and 38.3% burnout. Burnout was driven by low PA. No correlation was found between HCC and perceived stress, anxiety, depression, and burnout.

## Author Contributions

Conception and design: H.A., T.F., H.D., C.J., M.S.C. Data collection: H.A., H.D., C.J., G.G. Data analysis interpretation: H.A., T.F., M.S.C. All authors critically revised and approved the final manuscript.

## Conflicts of Interest

The authors declare no conflicts of interest.

## Supporting information


**Data S1.** aas70081‐sup‐0001‐Figures.

## Data Availability

The data that support the findings of this study are available on request from the corresponding author. The data are not publicly available due to privacy or ethical restrictions.
