# Supplementary material for: Physiological and Perceived Stress, Anxiety, Depression and Burnout Among ICU Staff During the COVID‐19 Pandemic in Sweden—An Observational Study
Source: Acta Anaesthesiol Scand. 2025 Jun 23;69(6):e70081. doi: 10.1111/aas.70081 (PMC12184077; doi:10.1111/aas.70081)
Supplement: Supplementary file 1 — Data S1. aas70081‐sup‐0001‐Figures. [file AAS-69-0-s001.docx]

**Supplementary figures**


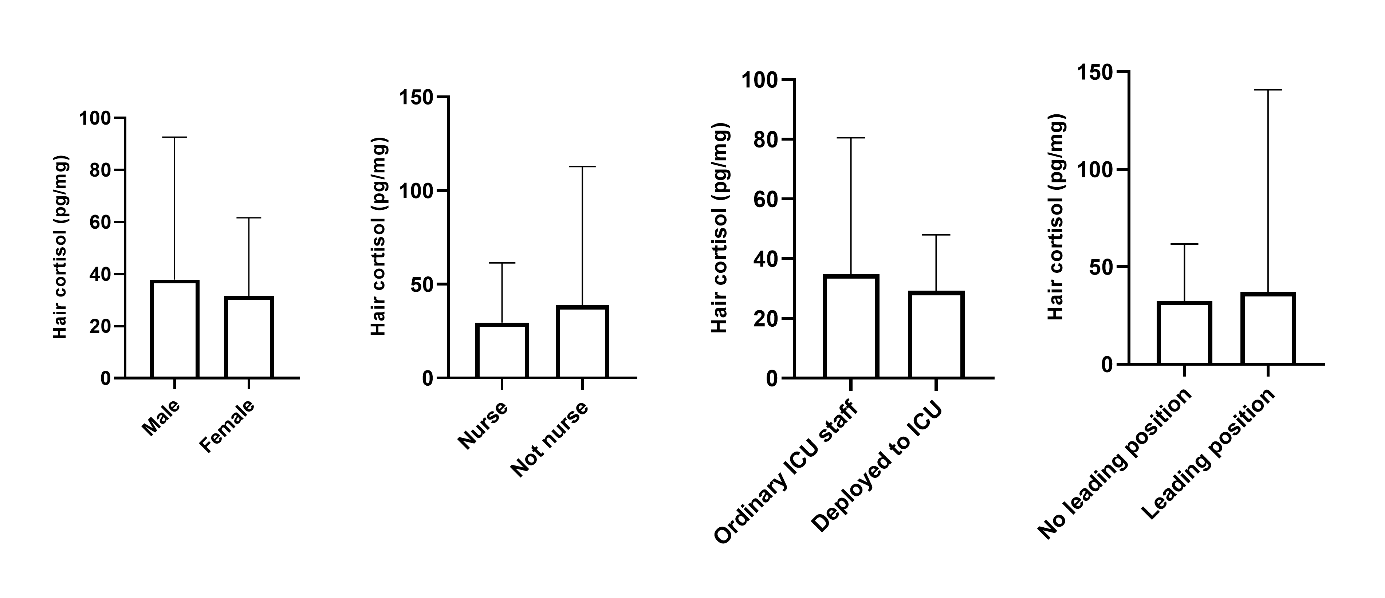


**Supplementary figure 1.** Hair cortisol concentrations stratified to sex, profession (nurse vs. not nurse), and ordinary place of work (ordinary vs deployed staff). n=274.


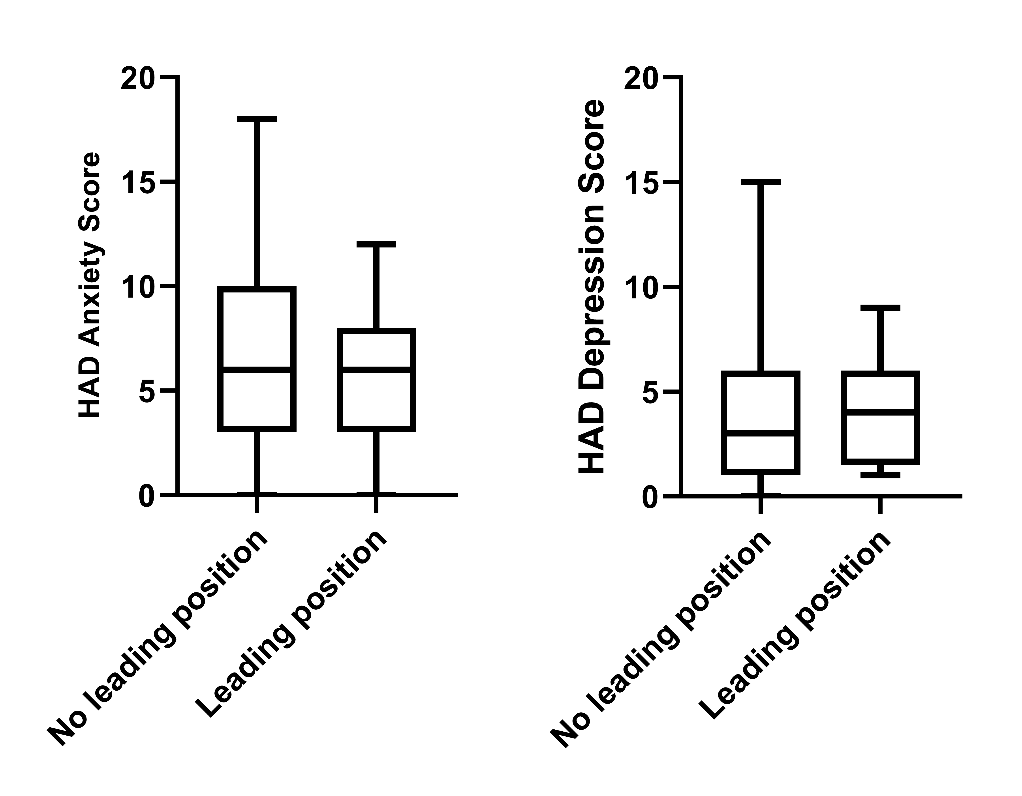


**Supplementary figure 2.** Anxiety and depression stratified to leading position. n=273.


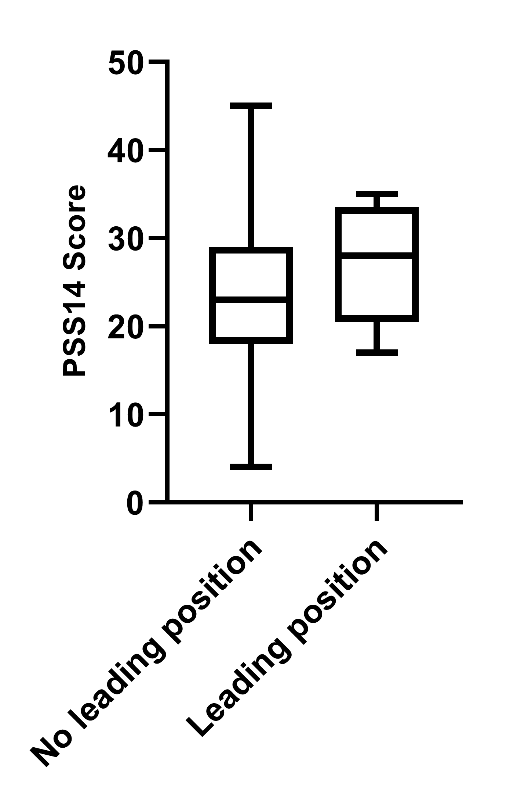


**Supplementary figure 3.** Perceived stress stratified to leading position. n=273.
